# Lung ultrasound B-lines, NT-proBNP, and left atrial volume Index for bedside characterization of preserved-ejection-fraction phenotype in acute heart failure: a single-center observational study

**DOI:** 10.3389/fcvm.2026.1877248

**Published:** 2026-06-05

**Authors:** Bin Peng, Yanxia Zhang

**Affiliations:** 1 Department of Cardiology, Xiangtan Central Hospital (Hunan University Affiliated Hospital), Xiangtan, Hunan, China; 2 Department of Emergency Medicine, University Health Center, Xiangtan University, Xiangtan, Hunan, China

**Keywords:** B-lines, decision curve analysis, HFpEF, left atrial volume index, lung ultrasound, NT-proBNP

## Abstract

**Background:**

Heart failure with preserved ejection fraction (HFpEF) is often difficult to identify at the bedside because no single clinical, biochemical, or echocardiographic marker is sufficiently definitive. Lung ultrasound B-lines, N-terminal pro-B-type natriuretic peptide (NT-proBNP), and left atrial volume index (LAVI) reflect different aspects of congestion and cardiac remodeling. This study evaluated whether combining these routinely available parameters could improve simplified bedside identification of HFpEF.

**Methods:**

This single-center prospective study included 220 patients hospitalized with acute decompensated heart failure at Xiangtan Central Hospital between January 2023 and December 2025. HFpEF was defined as left ventricular ejection fraction ≥ 50%, and patients with mildly reduced or reduced ejection fraction were classified as non-HFpEF. B-line score, NT-proBNP, LAVI, E/e′, body mass index, and atrial fibrillation status were recorded. Logistic regression, receiver operating characteristic curve analysis, and decision curve analysis were used to evaluate the performance and potential clinical utility of individual markers and combined models.

**Results:**

Among 220 patients, 57 had HFpEF and 163 had non-HFpEF. Individual markers showed limited-to-moderate discriminatory ability, with AUCs of 0.680 for B-line score, 0.655 for log-transformed NT-proBNP, and 0.550 for LAVI. The three-marker model combining B-line score, log-transformed NT-proBNP, and LAVI achieved an AUC of 0.737, with a sensitivity of 71.9% and specificity of 69.3%. The full exploratory model further increased the AUC to 0.765. Decision curve analysis suggested that the combined models provided greater net benefit than treat-all and treat-none strategies across a clinically relevant range of threshold probabilities.

**Conclusions:**

A combination of lung ultrasound B-lines, NT-proBNP, and LAVI showed moderate ability to distinguish patients with a preserved-ejection-fraction phenotype from those with lower LVEF among patients with acute heart failure. This approach should be interpreted as a bedside phenotype-characterization tool rather than a validated diagnostic model for confirming HFpEF.

## Introduction

Heart failure with preserved ejection fraction (HFpEF) accounts for a substantial proportion of heart failure cases and remains one of the most challenging phenotypes to identify in routine clinical practice. Unlike heart failure with reduced ejection fraction (HFrEF), HFpEF is characterized by preserved left ventricular ejection fraction but heterogeneous abnormalities involving diastolic dysfunction, left atrial remodeling, pulmonary congestion, vascular stiffness, and multiple extracardiac comorbidities. Current guidelines and diagnostic algorithms emphasize that HFpEF diagnosis should not rely on left ventricular ejection fraction alone, but should integrate symptoms, natriuretic peptides, and structural or functional evidence of cardiac abnormality (1–4).

In real-world bedside practice, however, simplified identification of HFpEF remains difficult. Symptoms such as dyspnea and edema are nonspecific, and natriuretic peptide levels may be influenced by age, renal function, obesity, and atrial fibrillation. Echocardiographic parameters such as left atrial volume index (LAVI) and E/e′ provide important information regarding chronic left atrial remodeling and elevated filling pressure, but these parameters alone may not fully capture active pulmonary congestion (5). Therefore, a clinically feasible approach that integrates pulmonary congestion, biochemical stress, and cardiac structural remodeling may improve bedside recognition of HFpEF.

Lung ultrasound (LUS) has emerged as a rapid, noninvasive, and repeatable tool for evaluating pulmonary congestion in patients with heart failure and may serve as an extension of bedside clinical examination in acute heart failure (6). B-lines reflect increased extravascular lung water and have been associated with natriuretic peptides, echocardiographic indices, and clinical outcomes in heart failure populations (7–9).

Recent studies have also suggested that B-lines are common in patients with preserved LVEF or HFpEF and may provide complementary information beyond conventional clinical and echocardiographic assessment. Importantly, patients with HFpEF may have no overt congestion at rest but develop pulmonary congestion during exercise; exercise-induced B-lines have been shown to occur together with worsening diastolic function and may improve the identification of exercise-induced increases in filling pressure (10, 11).

Therefore, this study aimed to characterize the preserved-ejection-fraction phenotype among patients hospitalized with acute heart failure and to explore whether lung ultrasound B-lines, NT-proBNP, and LAVI provide complementary bedside information for differentiating preserved-LVEF from non-preserved-LVEF phenotypes.

## Materials and methods

### Study design and population

This single-center prospective observational study was conducted at Xiangtan Central Hospital between January 2023 and December 2025. Consecutive patients hospitalized with acute decompensated heart failure were screened after admission. Patients were included if they had clinical symptoms or signs consistent with heart failure and underwent both transthoracic echocardiography and lung ultrasound during hospitalization. Heart failure was diagnosed according to contemporary guideline recommendations based on symptoms, signs, natriuretic peptide levels, and objective evidence of structural or functional cardiac abnormality (1, 2).

Patients were excluded if they had severe pulmonary disease that could substantially interfere with interpretation of lung ultrasound findings, including extensive pulmonary fibrosis, severe pneumonia, acute respiratory distress syndrome, or large pleural effusion. Patients with poor-quality echocardiographic or lung ultrasound images, acute coronary syndrome, hemodialysis, pregnancy, marked thoracic deformity, or inability to cooperate with ultrasound examination were also excluded.

The final analysis included 220 patients. According to left ventricular ejection fraction, patients were classified as HFpEF or non-HFpEF. For the purpose of this exploratory analysis, patients with acute heart failure were categorized according to LVEF into a preserved-ejection-fraction phenotype and a non-preserved-ejection-fraction phenotype. The preserved-ejection-fraction phenotype was defined as LVEF ≥ 50%, whereas the non-preserved-ejection-fraction phenotype included patients with mildly reduced or reduced LVEF. This LVEF-based classification was used for phenotype characterization and exploratory analysis, rather than as an independently adjudicated diagnosis of clinically definite HFpEF.

### Clinical and laboratory data collection

Baseline demographic and clinical data were collected from the electronic medical record system of Xiangtan Central Hospital, including age, sex, body mass index, hypertension, diabetes mellitus, coronary heart disease, and atrial fibrillation. Laboratory parameters included NT-proBNP, serum creatinine, estimated glomerular filtration rate, and other routinely available biochemical indices. Echocardiographic and lung ultrasound parameters included LVEF, LAVI, E/e′, and B-line score. Because NT-proBNP typically has a right-skewed distribution, log-transformed NT-proBNP was used in regression and receiver operating characteristic analyses.

Admission vital signs, including heart rate, blood pressure, oxygen saturation, and respiratory rate, were not systematically recorded in the original study database and were therefore not included in the main analysis.

### Echocardiographic assessment

Transthoracic echocardiography was performed by experienced physicians using standard protocols. The following echocardiographic parameters were recorded: left ventricular ejection fraction, left atrial volume index, E/e′, left ventricular end-diastolic dimension, left ventricular end-systolic dimension, systolic pulmonary artery pressure, and inferior vena cava diameter. Left ventricular ejection fraction was used to define HF phenotype. LAVI was selected as a representative structural marker of chronic left atrial remodeling, while E/e′ was used as a noninvasive functional marker related to left ventricular filling pressure (3–5).

### Lung ultrasound assessment

Lung ultrasound was performed using a standardized 8-zone protocol. Bilateral anterior and lateral chest regions were examined, including two anterior and two lateral zones on each hemithorax. Examinations were performed with patients in a semi-recumbent or sitting position whenever clinically feasible. B-lines were defined as vertical hyperechoic reverberation artifacts arising from the pleural line, extending to the bottom of the screen without fading, and moving synchronously with lung sliding. The total B-line score was calculated as the sum of B-lines across all eight zones, with higher scores indicating greater pulmonary congestion. When B-lines were confluent, the number of B-lines was estimated according to the proportion of the intercostal space occupied by B-lines. The 8-zone protocol was selected because it is feasible in hospitalized patients with acute heart failure and has been used in previous heart failure studies (6, 8, 12).

### Outcome definition

The main exploratory comparison was between patients with preserved LVEF and those with mildly reduced or reduced LVEF. The main candidate predictors were B-line score, log-transformed NT-proBNP, and LAVI. Additional variables considered in exploratory models included E/e′, body mass index, and atrial fibrillation.

### Statistical analysis

Continuous variables were expressed as mean ± standard deviation or median with interquartile range, and categorical variables as numbers and percentages. Between-group comparisons were performed using the Student's t test or Mann–Whitney U test for continuous variables and the chi-square test or Fisher's exact test for categorical variables, as appropriate.

The primary outcome was HFpEF status, with HFpEF coded as 1 and non-HFpEF coded as 0. NT-proBNP was log-transformed because of its skewed distribution. Logistic regression was used to evaluate associations between candidate variables and the preserved-LVEF phenotype. A three-marker model including B-line score, log-transformed NT-proBNP, and LAVI was constructed. A full exploratory model additionally included E/e′, body mass index, and atrial fibrillation. These variables were selected based on clinical relevance and their common use in HFpEF diagnostic frameworks, particularly the H2FPEF score. A sensitivity analysis additionally including age was performed to evaluate whether age materially influenced the performance of the full exploratory model. Odds ratios and 95% confidence intervals were reported.

Receiver operating characteristic curve analysis was used to assess discriminatory performance. The area under the curve, optimal cutoff value based on the Youden index, sensitivity, and specificity were calculated. Decision curve analysis was performed to evaluate the potential clinical net benefit of the combined models. All tests were two-sided, and *P* < 0.05 was considered statistically significant. Analyses were performed using R software, version 4.3.2.

## Results

**Figure 1:**
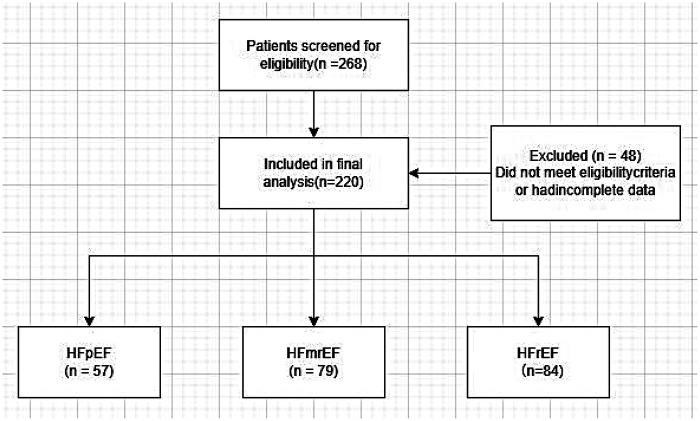
Study flowchart of patient selection and classification.

A total of 268 patients hospitalized with acute decompensated heart failure were screened. After excluding 48 patients who did not meet the eligibility criteria or had incomplete data, 220 patients were included in the final analysis. Among them, 57 patients were classified as HFpEF and 163 were classified as non-HFpEF, including 79 patients with HFmrEF and 84 patients with HFrEF. The patient selection process is shown in Figure 1.

Baseline clinical and imaging characteristics are summarized in Table 1. Patients with HFpEF tended to have a higher body mass index, a higher prevalence of atrial fibrillation, lower B-line score, and lower NT-proBNP levels than patients with non-HFpEF. Median B-line score was 20.9 in HFpEF, 25.1 in HFmrEF, and 27.9 in HFrEF. Median NT-proBNP level was 6,236.8 pg/mL in HFpEF, 9,426.2 pg/mL in HFmrEF, and 14,543.2 pg/mL in HFrEF. LAVI showed substantial overlap among the three phenotypes, with median values of 42.3 mL/m² in HFpEF, 39.3 mL/m² in HFmrEF, and 36.3 mL/m² in HFrEF.

**Table 1:** Baseline clinical and imaging characteristics according to heart failure phenotype.

Variable	HFrEF (*n* = 84)	HFmrEF (*n* = 79)	HFpEF (*n* = 57)	*P* value
Age, years	70.3 ± 10.1	72.5 ± 10.0	71.1 ± 8.8	0.365
Male sex, *n* (%)	58 (69.0)	37 (46.8)	24 (42.1)	0.002
Body mass index, kg/m²	24.0 ± 3.5	23.4 ± 3.1	24.8 ± 4.0	0.067
Atrial fibrillation, *n* (%)	20 (23.8)	19 (24.1)	24 (42.1)	0.033
Hypertension, *n* (%)	33 (39.3)	35 (44.3)	35 (61.4)	0.030
B-line score	27.9 (23.4–36.0)	25.1 (20.9–31.1)	20.9 (16.2–27.3)	<0.001
NT-proBNP, pg/mL	14,543.1 (7,445.4–20,572.5)	9,426.2 (5,587.8–14,798.2)	6,236.8 (4,003.3–12,672.1)	<0.001
LAVI, mL/m²	36.2 (25.8–47.5)	39.3 (30.0–51.0)	42.3 (26.7–54.1)	0.245
E/e′	16.2 (11.6–19.3)	14.2 (11.1–18.1)	15.4 (12.1–18.6)	0.247
LVEF, %	32.1 ± 5.1	43.9 ± 2.4	57.2 ± 5.8	<0.001

Data are presented as mean ± SD, median (IQR), or *n* (%). Between-group comparisons used ANOVA, Kruskal–Wallis test, chi-square test, or Fisher exact test as appropriate.

In the three-marker model including B-line score, log-transformed NT-proBNP, and LAVI, B-line score and log-transformed NT-proBNP were independently associated with HFpEF. B-line score was inversely associated with HFpEF (OR 0.94, 95% CI 0.90–0.97, *P* < 0.001), and log-transformed NT-proBNP was also inversely associated with HFpEF (OR 0.60, 95% CI 0.41–0.89, *P* = 0.010). LAVI showed a positive but nonsignificant association with HFpEF (OR 1.01, 95% CI 1.00–1.04, *P* = 0.142). In the full exploratory model including B-line score, log-transformed NT-proBNP, LAVI, E/e′, body mass index, and atrial fibrillation, B-line score (OR 0.94, 95% CI 0.90–0.97, *P* = 0.001), log-transformed NT-proBNP (OR 0.54, 95% CI 0.36–0.81, *P* = 0.003), body mass index (OR 1.15, 95% CI 1.04–1.27, *P* = 0.008), and atrial fibrillation (OR 2.25, 95% CI 1.11–4.54, *P* = 0.024) remained associated with HFpEF. LAVI and E/e′ were not statistically significant in the fully adjusted model. The logistic regression results are presented in Table 2.

**Table 2:** Logistic regression models for identifying HFpEF.

Model	Variable	OR	95% CI	*P* value
Three-marker model	B-line score	0.94	0.90–0.97	<0.001
	log NT-proBNP	0.60	0.41–0.89	0.010
	LAVI, mL/m²	1.01	1.00–1.04	0.142
Full exploratory model	B-line score	0.94	0.90–0.97	0.001
	log NT-proBNP	0.54	0.36–0.81	0.003
	LAVI, mL/m²	1.02	1.00–1.04	0.070
	E/e′	1.01	0.95–1.09	0.685
	Body mass index	1.15	1.04–1.27	0.008
	Atrial fibrillation	2.25	1.11–4.54	0.024

OR, odds ratio; CI, confidence interval; LAVI, left atrial volume index; NT-proBNP, N-terminal pro-B-type natriuretic peptide.

**Figure 2:**
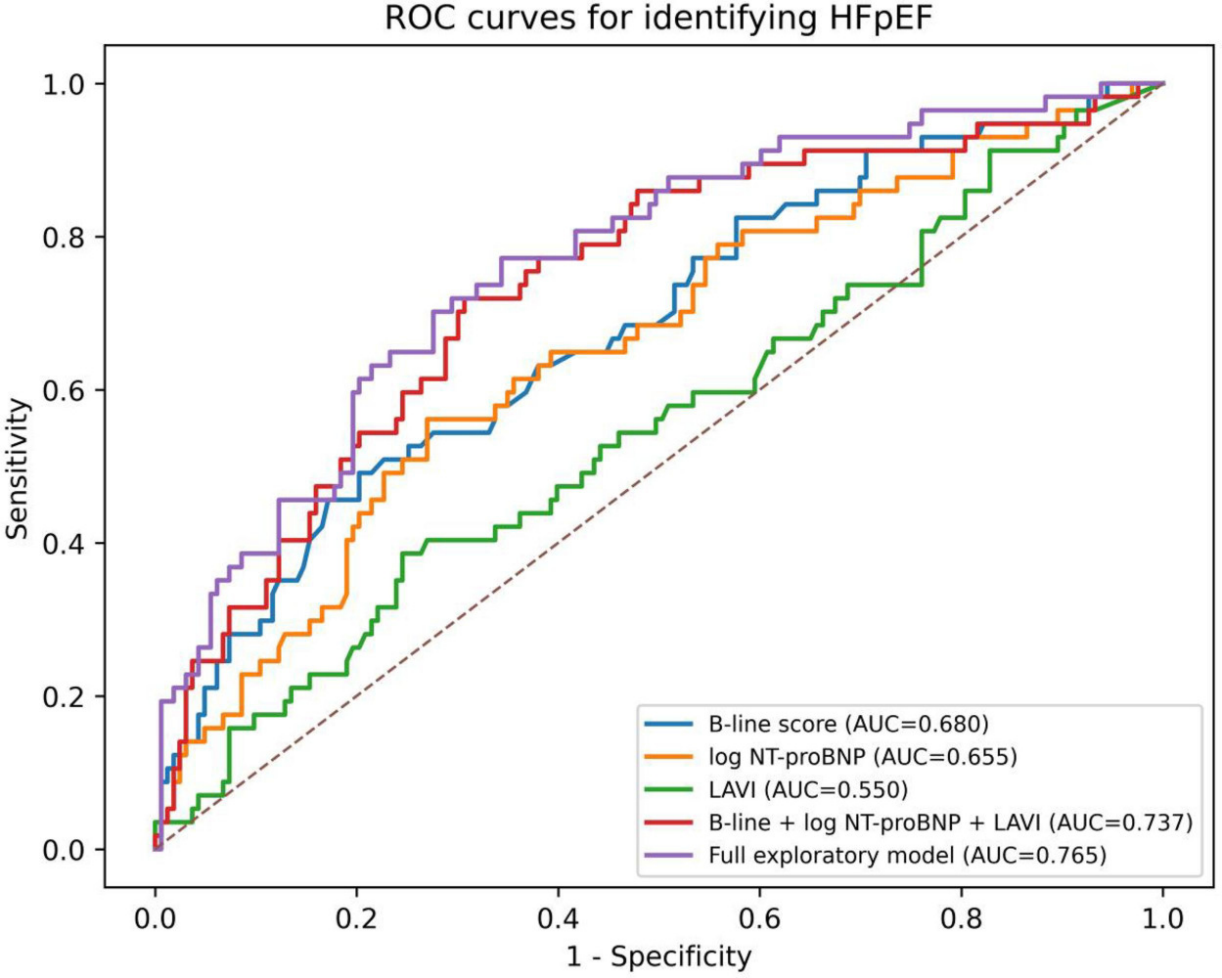
Receiver operating characteristic curves for individual markers and combined models in identifying HFpEF.

The discriminatory performance of individual markers and combined models is shown in Table 3 and Figure 2. Individual markers showed limited-to-moderate performance for identifying HFpEF, with AUCs of 0.680 for B-line score, 0.655 for log-transformed NT-proBNP, 0.550 for LAVI, 0.522 for E/e′, 0.581 for body mass index, and 0.591 for atrial fibrillation. The three-marker model combining B-line score, log-transformed NT-proBNP, and LAVI achieved an AUC of 0.737. At the optimal cutoff based on the Youden index, the model showed a sensitivity of 71.9% and a specificity of 69.3%. The full exploratory model, which additionally included E/e′, body mass index, and atrial fibrillation, showed a slightly higher AUC of 0.765, with a sensitivity of 77.2% and a specificity of 65.6% at the optimal cutoff. In the sensitivity analysis additionally including age, model discrimination was essentially unchanged compared with the primary full exploratory model (AUC 0.766 vs. 0.765), and age was not independently associated with the preserved-LVEF phenotype (OR 0.994, 95% CI 0.958–1.030, *P* = 0.725).

**Table 3:** Diagnostic performance of individual markers and combined models for identifying HFpEF.

Model	AUC (95% CI)	Optimal cutoff^a^	Sensitivity, %	Specificity, %
B-line score	0.680 (0.600–0.763)	0.325	49.1	79.8
log NT-proBNP	0.655 (0.575–0.733)	0.284	56.1	73.0
LAVI	0.550 (0.460–0.640)	0.278	38.6	75.5
E/e′	0.522 (0.445–0.597)	0.256	64.9	45.4
Body mass index	0.581 (0.484–0.668)	0.237	75.4	41.1
Atrial fibrillation	0.591 (0.525–0.659)	0.378	42.1	76.1
B-line + log NT-proBNP + LAVI	0.737 (0.664–0.808)	0.250	71.9	69.3
Full exploratory model	0.765 (0.692–0.838)	0.235	77.2	65.6

AUC, area under the receiver operating characteristic curve.

a Cutoff values are probability thresholds derived from logistic regression-predicted probabilities.

**Figure 3:**
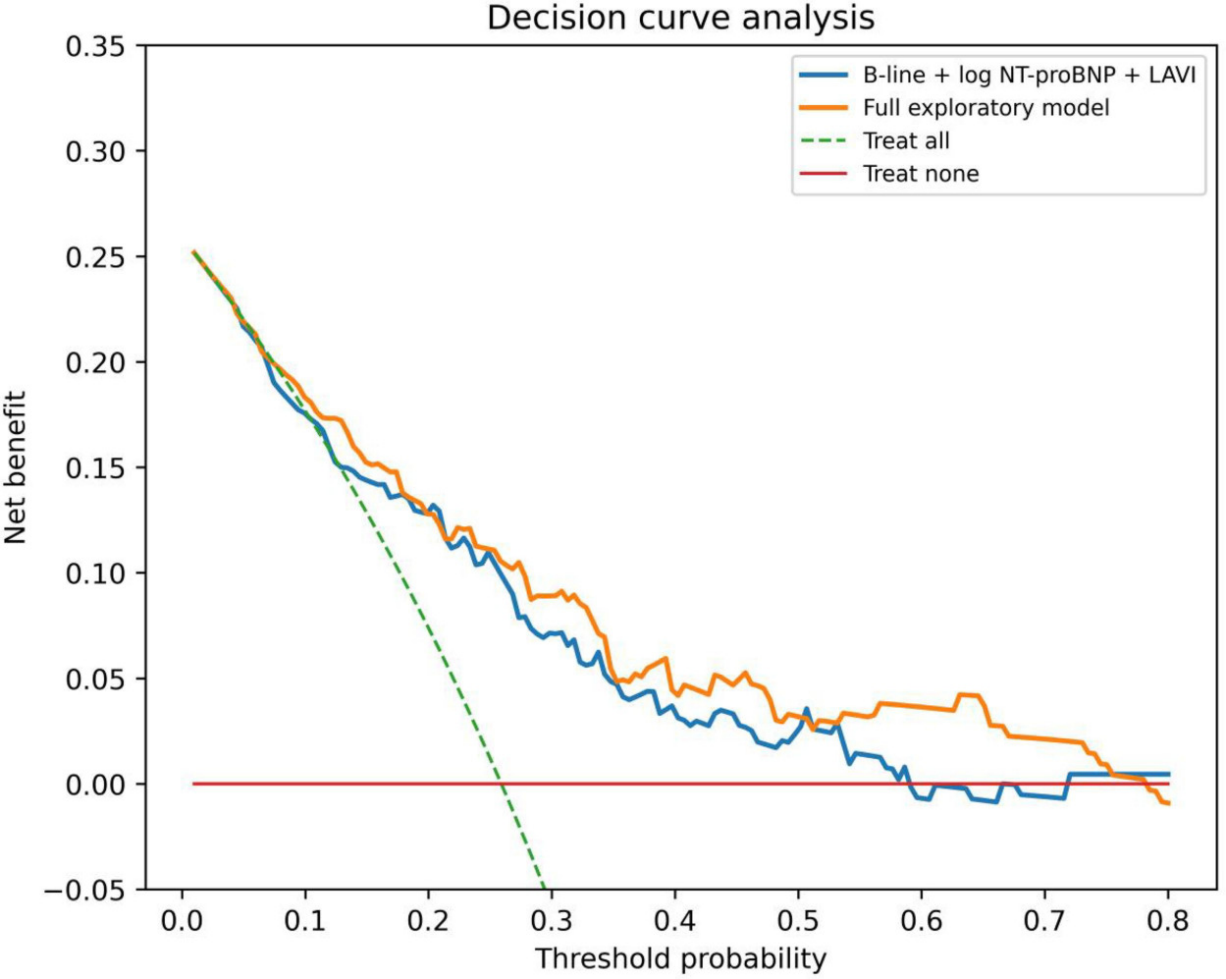
Decision curve analysis of the three-marker model and full exploratory model.

Decision curve analysis demonstrated that the three-marker model and the full exploratory model provided greater net benefit than the treat-all and treat-none strategies across a clinically relevant range of threshold probabilities (Figure 3). The full exploratory model showed a modestly higher net benefit than the three-marker model over part of the threshold range; however, the difference was not large, suggesting that the simpler three-marker model may be more practical for bedside use.

Overall, no single marker was sufficient for reliable identification of HFpEF. The combination of B-line score, log-transformed NT-proBNP, and LAVI improved discriminatory performance to an acceptable range, while the addition of E/e′, body mass index, and atrial fibrillation provided only modest further improvement. These findings support the potential value of a simplified multimodal bedside approach for HFpEF identification.

## Discussion

In this single-center prospective study, we evaluated the value of combining lung ultrasound B-lines, NT-proBNP, and LAVI for simplified bedside identification of HFpEF among patients hospitalized with acute decompensated heart failure. The main findings were as follows. First, individual markers showed only limited-to-moderate discriminatory ability for identifying HFpEF. Second, the three-marker model combining B-line score, log-transformed NT-proBNP, and LAVI improved the AUC to an acceptable range. Third, adding E/e′, body mass index, and atrial fibrillation provided only modest additional improvement. Finally, decision curve analysis suggested that the combined models may provide greater clinical net benefit than treat-all and treat-none strategies across a clinically relevant threshold range. These findings support the potential value of a simplified multimodal bedside approach for HFpEF identification (13).

The diagnostic difficulty of HFpEF is largely related to its heterogeneous pathophysiology. HFpEF is not a single disease entity, but a clinical syndrome involving diastolic dysfunction, left atrial remodeling, systemic inflammation, vascular stiffness, obesity, atrial fibrillation, renal dysfunction, and other comorbid conditions (14, 15). Therefore, relying on a single parameter is unlikely to provide sufficient diagnostic confidence. In this context, the three markers used in our model represent different but complementary dimensions of HFpEF assessment. B-lines reflect pulmonary congestion, NT-proBNP reflects myocardial wall stress, and LAVI reflects chronic left atrial remodeling. Although each individual marker showed limited discriminatory performance in our study, their combination improved discrimination, suggesting that integration of pulmonary, biochemical, and structural information may better reflect the complex HFpEF phenotype than any single marker alone.

An important observation was that patients with the preserved-LVEF phenotype had lower B-line scores and lower log-transformed NT-proBNP levels than those with mildly reduced or reduced LVEF. This finding is clinically plausible because patients with reduced LVEF often present with more prominent systolic dysfunction, ventricular remodeling, neurohormonal activation, and resting pulmonary congestion during acute decompensation. However, lower resting NT-proBNP and B-line burden should not be interpreted as the absence of clinically relevant congestion in preserved-LVEF patients. Preserved-LVEF heart failure is a heterogeneous phenotype, and symptoms may be driven by diastolic dysfunction, left atrial pressure elevation, atrial fibrillation, obesity, renal dysfunction, and exercise-induced increases in filling pressure. In particular, some patients with HFpEF may have limited congestion at rest but develop pulmonary congestion during exercise, and obesity may also attenuate natriuretic peptide levels. Therefore, B-lines and NT-proBNP should be interpreted as complementary markers within an integrated clinical and echocardiographic assessment rather than as isolated indicators of HFpEF severity (16). However, HFpEF should not be interpreted as a low-congestion phenotype. Prior studies have shown that pulmonary congestion assessed by lung ultrasound is common in patients with HFpEF and may provide clinically relevant information beyond symptoms and routine physical examination (17, 18). Thus, the role of B-lines in HFpEF may be better understood as a complementary bedside congestion marker rather than an isolated diagnostic test.

The full exploratory model achieved only modest improvement over the three-marker model. Although body mass index and atrial fibrillation were associated with HFpEF, the increase in AUC was limited. This is clinically important because a more complex model may not always be preferable if it provides only marginal improvement in discrimination. Obesity and atrial fibrillation are well-recognized contributors to the HFpEF phenotype and may also affect natriuretic peptide interpretation, which further supports the need for integrated assessment (16). In bedside practice, a simpler model based on B-lines, NT-proBNP, and LAVI may therefore be easier to apply and interpret. The decision curve analysis further suggested that the combined models may provide a higher net benefit than treat-all and treat-none strategies over a relevant range of threshold probabilities, supporting their potential clinical utility (19). Nevertheless, the overall discriminatory performance remained moderate rather than excellent, indicating that this approach should be viewed as an adjunctive tool rather than a replacement for guideline-based diagnostic evaluation.

Several limitations should be acknowledged. First, it should be emphasized that our analysis was not intended to confirm HFpEF diagnosis using B-lines, NT-proBNP, and LAVI. Because HFpEF was not adjudicated by invasive hemodynamics, exercise testing, expert consensus, or formal HFA-PEFF/H2FPEF classification, the present findings should be interpreted as characterizing a preserved-LVEF acute heart failure phenotype rather than establishing a validated diagnostic model for HFpEF. Second, this was a single-center study, which may limit the generalizability of the findings. Third, although the sample size was acceptable for exploratory analysis, the number of patients with preserved LVEF was still relatively limited. Fourth, external validation was not performed, and the stability of the model should be tested in independent cohorts. Fifth, admission vital signs and respiratory support details, including heart rate, blood pressure, oxygen saturation, respiratory rate, and oxygen therapy status, were not systematically recorded in the original study database. Therefore, their potential influence on B-line burden, natriuretic peptide levels, and LVEF phenotype classification could not be fully evaluated. Finally, lung ultrasound assessment may be influenced by operator experience and noncardiac pulmonary conditions, although standardized scanning procedures were used.

## Data Availability

The raw data supporting the conclusions of this article will be made available by the authors, without undue reservation.
